# Exploring the ethical issues posed by AI and big data technologies in drug development

**DOI:** 10.3389/fpubh.2025.1585180

**Published:** 2025-10-20

**Authors:** Yulu Fan, Yancheng Wu, Zitong Wang

**Affiliations:** ^1^College of Pharmacy, Shanghai University of Medicine & Health Science, Shanghai, China; ^2^Shanghai University of Medicine & Health Sciences’ Active Health for Everyone Science Popularization and Collaborative Governance Platform, Shanghai, China; ^3^Shanghai University of Medicine & Health Sciences, The Center of Policy Research and Safety Evaluation for Medical Devices, Shanghai, China; ^4^School of Health Science and Engineering, University of Shanghai for Science and Technology, Shanghai, China; ^5^Digital and Intelligent Empowerment Biomedical Innovation Center, School of Pharmacy, Shanghai University of Medicine & Health Sciences, Shanghai, China

**Keywords:** artificial intelligence, big data, drug development, ethical issues, responsible innovation

## Abstract

The rapid development and wide application of Artificial Intelligence (AI) and Big Data technologies have profoundly changed the way industries around the world operate, from finance, transportation, education to media, the integration of the two not only improves the efficiency of the industry, but also optimizes the quality of service and decision-making process to a large extent. In the era of deep integration of Biomedicine and AI, AI and Big Data technology are reconstructing the paradigm of drug development with unprecedented intensity. The long cycle of traditional drug development, which takes a decade and billions of dollars in investment, is being compressed to 2 years or even less under the drive of AI. Through big data analytics and deep learning techniques, AI can greatly improve R&D efficiency and accuracy in a variety of aspects such as compound screening, efficacy prediction, and clinical (pre) experiment design. However, the use of AI and big data in drug discovery and development also raises corresponding ethical issues, such as data privacy protection and algorithmic transparency. This article will systematically analyze the technological breakthroughs, potential risks, and governance paths of AI and big data in drug development. It will explore how to strengthen the bottom-line of safety and ethics in the Efficiency Revolution and build a responsible innovation ecosystem.

## 1 Introduction

As a core field for improving human health, drug research and development is undergoing an efficiency revolution driven by AI technology, but the current data controversies reveal the contradiction between technological acceleration and ethics. Based on an in-depth analysis of the ethical challenges in drug R&D, this paper constructs an ethical evaluation framework centered on autonomy, justice, non-maleficence, and beneficence, and, through the three evaluation dimensions of data-mining informed consent, pre-clinical dual-track verification, and transparency in patient recruitment, systematically dissects the ethical risks across the entire AI R&D cycle. Finally, it puts forward referential strategies, including strengthening ethical supervision and regulation of AI algorithms, improving data-privacy protection, enhancing algorithmic openness and transparency, building accountability mechanisms, reinforcing long-cycle monitoring of AI technology in drug R&D (1), and encouraging multi-party participation and informed consent from patients and the public (2). By implementing these responsible-innovation strategies, the rights and interests of subjects can be more effectively protected, the safety and effectiveness of drugs ensured, and fair drug distribution and accessibility promoted. The integrated application of these ethical principles and innovation strategies is expected to push drug R&D toward a more responsible future.

## 2 Application of AI and big-data technology in the entire cycle of drug research and development

### 2.1 Overview of the ethical-evaluation theoretical framework for AI application throughout the drug-development cycle

Based on ethical-evaluation principles, from the initiation of AI technology in drug R&D to post-marketing surveillance, this paper realizes ethical-compliance control of AI applications through phased risk mapping, comprehensively evaluating the benefits and risks of this technology across the whole drug-development process. The current AI ethical framework is founded on universal ethical principles, with four core principles: autonomy (respect for individual autonomy, e.g., informed consent), justice (avoiding bias and discrimination, ensuring fairness in resources and opportunities), non-maleficence (avoiding potential risks and harms), and beneficence (promoting social well-being). Three evaluation dimensions corresponding to the three research stages are constructed—requirements for informed consent in the data-mining stage, a dual-track verification mechanism in pre-clinical research, and transparency requirements in the patient-recruitment stage. The paper systematically analyzes the technological breakthroughs of AI and big data in drug R&D and deeply examines typical risk points such as privacy leakage of group genetic data, undetected intergenerational toxicity, and algorithmic bias leading to unfair enrollment, aiming to regulate the ethical boundaries of AI technology in application and balance technological innovation with risk prevention and control.

The ethical-evaluation framework constructed herein is highly aligned with the current core principles of AI ethics: it emphasizes “informed consent in the data-mining stage,” requiring that the purpose of genetic-data collection be explicitly stated, directly echoing the core requirement of “respect for individual autonomy”; it focuses on “transparency in patient recruitment,” implementing the justice principle of “avoiding discrimination and ensuring fairness” by detecting algorithmic bias and opposing geographical bias in clinical trials; it proposes a “pre-clinical dual-track verification mechanism,” requiring that AI virtual-model predictions be synchronously combined with actual animal experiments to avoid the omission of long-term toxicity due to shortened R&D cycles, directly corresponding to the core of “avoiding harm”; the overall goal is to ensure, through ethical norms, that AI technology improves drug-development efficiency while ultimately serving human health, in line with the beneficence requirement of “promoting well-being.”

However, the current AI ethical framework mostly consists of abstract principles; this paper expands it into a concrete operational system for the entire cycle of drug R&D: the general principles are disassembled into the three-dimensional evaluation of “data mining — pre-clinical — patient recruitment,” each dimension corresponding to quantifiable operational standards, turning abstract principles into executable processes; in light of the particularities of drug development, it supplements areas not covered by the general framework.

By analyzing real problems, this paper also indirectly criticizes the deficiencies of the current AI ethical framework in practice. Domestically, institutional gaps exist in cross-border transmission of group genetic data and cross-regional verification of clinical-trial data; the current framework lacks supporting regulatory rules for addressing specific risks of AI technology in drug R&D. International cooperation faces fragmented ethical standards and ambiguous divisions of responsibility, exposing the framework’s inadequacies in a global context; the chain of “historical data bias — algorithm amplification — clinical injustice” in drug R&D is insufficiently considered, and algorithm-audit mechanisms need to be strengthened.

### 2.2 Application of AI technology in drug research and development

In the field of drug development, AI technology is bringing disruptive changes. Demis Hassabis, CEO of Google DeepMind and winner of the 2024 Nobel Prize in Chemistry, once pointed out that traditional drug development takes an average of 10 years and costs billions of dollars, whereas AI technology is expected to greatly shorten this process. This view vividly demonstrates the tremendous potential of AI to accelerate drug-development progress.

Artificial intelligence has significantly improved R&D efficiency and precision by optimizing drug-discovery processes and clinical-trial design, and this breakthrough stems from two core advantages of AI. AI technology replaces laboratory operations with virtual screening, transferring traditional bottle-and-flask compound screening into computer simulations, optimizing trial design and improving the scientificity of decision-making. For example, the cooperation between Iktos and Pfizer accelerated the discovery of small-molecule drugs through AI, enriching the drug-candidate library with AI-designed compounds. It is worth noting that, in the AI-accelerated drug-discovery process, the evaluation dimension of informed consent in the data-mining stage can be incorporated. For instance, the cooperation between Insitro and Gilead, which developed predictive models with AI technology to identify new drug targets (3), not only improves the speed of target discovery but may also reduce R&D costs. Insitro explicitly informed subjects of the purpose of data collection involving group genetic data in accordance with the framework—contrasting with the ethical controversy caused by ambiguous consent forms in DeepMind’s NHS data sharing.

Animal experiments are a key link in verifying the safety and effectiveness of drugs, but traditional methods are characterized by low efficiency and long cycles. The application of AI technology in simulating animal physiological responses provides a new solution to this dilemma. In traditional mouse experiments, second- and third-generation studies often require a great deal of time to collect data. With AI technology, researchers can use existing genetic data and biological knowledge to build virtual mouse intergenerational models that simulate the physiological characteristics and drug responses of offspring mice under different genetic combinations, greatly shortening research intervals and accelerating drug-development progress. In this process, however, the “pre-clinical dual-track verification mechanism” requires that traditional animal experiments be retained as controls to avoid the limitations of extrapolating from animal models, as in the thalidomide incident.

### 2.3 Retrospective value of big-data technology

Big-data technology plays a key role in modern drug R&D; it not only accelerates the speed of drug discovery and testing but also improves research quality and accuracy (4). By analyzing massive genetic datasets, researchers can identify gene variants related to specific diseases, providing clues for the development of targeted therapies. For example, Gaussian Process Regression (GPR) models are used to predict the bioactivity of molecules (5), helping decision-making in drug design. In the clinical-trial stage, big data optimizes trial design by analyzing historical trial data, improving the efficiency and adaptability of clinical trials (6). In personalized medicine, by analyzing patients’ genetic data and lifestyle information, individualized treatment plans can be tailored for each patient. Tools such as DeepChem (7) and the BRENDA database (8) support compound-toxicity prediction and enzyme-activity research, while Recursion Pharmaceuticals promotes new-drug discovery by using machine learning to analyze cellular phenotypic changes (9). These applications show how big-data technology promotes every stage from drug discovery to market, making disease treatment more precise and efficient.

Looking back at drug-development history, the “thalidomide incident” is a typical example of the shortcomings of traditional drug-safety evaluation. The use of thalidomide caused more than 12,000 babies worldwide to suffer severe outcomes such as limb deformities. If modern big-data analytical capabilities had been available at that time, risk-prediction models built by combining natural-language processing of medical-record texts with machine-learning algorithms could have quickly locked onto the association between thalidomide and infant deformities, greatly increasing drug-harm traceability efficiency and avoiding more tragedies. This retrospective big-data analysis provides a new path for mining potential associations in drug-harm events and improving drug-safety research.

Although big data shows strong power in retrospective research, prospective drug development still relies on a strict clinical-trial system. The correlations revealed by big-data analysis cannot directly establish causality; for example, data show a correlation between caffeine intake during pregnancy and infant health problems, but other confounding factors may interfere. Clinical trials, through randomized-controlled designs and double-blind experiments, verify causal mechanisms of drug efficacy and safety in controlled environments, providing decisive evidence for drug approval. Thus, big-data analysis can serve as an important auxiliary tool, complementing clinical trials and jointly consolidating the scientificity and reliability of drug R&D.

### 2.4 Drug-development model combining AI and big data

In modern drug R&D, the combination of AI and big data has significantly improved drug-development efficiency and accuracy (10). Specifically, the cooperation between GSK and Exscientia (11) optimizes drug-molecule design with AI technology, predicting molecular-structural activity for specific biological targets and iteratively optimizing with big data, greatly shortening the discovery cycle of drug-candidate molecules (12). For example, Exscientia reported that its AI system successfully designed a new obsessive-compulsive disorder drug candidate in less than a year, whereas traditional methods usually need many years (13). In addition, cooperation between Pfizer and IBM Watson Health also shows how AI and big data accelerate biomarker discovery through the Watson for Drug Discovery platform (14), which is important for new-target discovery, new-drug development, and personalized medicine. These cases not only prove that AI and big data can increase R&D speed but, through precise analysis, can optimize the entire R&D process, improving the success rate and economic efficiency of drug development. This technical integration represents a main developmental trend in current drug R&D, and its application potential will continue to expand.

## 3 Ethical risks of AI technology in accelerated drug R&D

### 3.1 Ethical risks inherent in algorithms

#### 3.1.1 Ethical breach of the pre-clinical dual-track verification mechanism

Within the safety-evaluation system of drug R&D, inherent differences between animal models and human physiology constitute a biological barrier that AI-simulation technology cannot surmount; this belongs to the technical maladaptation of algorithms to biological complexity. Taking thermoregulation as an example, the core body temperature of chickens is maintained at 29–30 °C, fundamentally different from the human metabolic environment at 37 °C; this temperature gap markedly affects the activity expression of drug-metabolizing enzymes, causing divergence between metabolic rates and toxicological characteristics of drugs in low-temperature animals and the actual human response. Similarly, the hepatic UGT1A1 enzyme activity of rodents is only one-tenth that of humans, so drugs such as irinotecan show far lower toxicity in animal experiments than in clinical observation, exposing significant interspecies differences in metabolic pathways.

The limitations of in-vitro experimental systems are even more prominent: the cell-culture environment lacks the complex in-vivo tissue microenvironment, so doxorubicin shows cardiac toxicity *in vitro* 30–50% lower than in animal experiments. Although AI technology can integrate multi-species data by constructing physiologically based pharmacokinetic (PBPK) models, its predictive accuracy is still limited by the completeness of underlying biological data—for example, differences between rhesus monkeys and humans in the IL-6 cytokine-signaling pathway cause antibody drugs targeting this pathway to show remarkable efficacy in animal experiments but to be forced to terminate in Phase I due to cytokine storm.

This biological-difference simulation dilemma essentially reflects the “data-dependence” of AI models. When training data lack deep annotation of cross-species physiological characteristics, even algorithms optimized through transfer learning cannot accurately predict the dynamic response of drugs in the complex human physiological environment. Therefore, AI-simulated rat intergenerational experiments must synchronously retain chronic-toxicity tests in non-rodent animals—this is the concrete implementation of the principle of “non-maleficence.”

#### 3.1.2 Ethical absence of the transparency requirement in patient recruitment

When using AI and big-data technology in drug R&D, one must be alert to data and algorithm bias. Such bias often exists in multiple links—data collection, processing, and algorithm design—and, in severe cases, may threaten the clinical safety and effectiveness of drugs. Data on which drug R&D relies must cover a broad population to ensure that the developed drug can benefit all patients. However, historically, participants in clinical trials have often lacked diversity; e.g., certain races or specific groups are under-represented, which may cause AI algorithms to predict effects inaccurately for these groups. In such cases, AI trained on imperfect data may show bias in race, gender, age, etc., thus affecting the fairness and effectiveness of drug R&D (15). A study evaluating sex, racial, and ethnic differences in U. S. cardiovascular trials found that African-American and Hispanic participants were significantly under-represented in key clinical trials, especially coronary-artery bypass graft (CABG) trials, where their participation rates were far lower than those of whites (16). Such imbalance may bias predictive models because models are usually trained on the population of clinical trials.

Besides the lack of diversity among participants, algorithm design itself may harbor bias (17). AI systems generally learn historical data to predict future outcomes; if historical data contain bias, the algorithms will amplify it. Moreover, developers’ subjectivity may inadvertently affect fairness (18). A 2019 study found that an algorithm used to predict patients’ future health risk showed significant predictive bias among different races. The algorithm took medical cost as a proxy for health need, but because Black patients incurred lower costs, it incorrectly assessed them as healthier than white patients with the same condition. Correcting this bias could raise the proportion of Black patients receiving additional care from 17.7 to 46.5% (19).

#### 3.1.3 Safety and credibility issues

With the widespread use of AI and big-data technology, challenges in safety and credibility are increasingly prominent. The credibility problem of AI and big data in drug R&D must also consider data timeliness and relevance (20). Over time, population health status and disease spectra may change; if the data used in AI training do not reflect these changes, the developed drugs may not effectively address current health challenges. Furthermore, different regions and environments may differently influence diseases, requiring AI models to recognize and adapt to such diversity to avoid “one-size-fits-all” solutions. A study showed that, in HIV/AIDS drug R&D, a major issue is the geographic and genetic diversity among viral strains: different HIV subtypes may tend to develop higher resistance to certain drugs (21), posing new challenges to drug development.

Moreover, safety is one of the most critical considerations in drug R&D. AI models rely on large data inputs and complex algorithms when designing drugs and predicting drug interactions. If input data are of poor quality or incomplete, AI-recommended drugs or treatment plans may carry potential safety risks. IBM’s Watson, initially highly anticipated for providing innovative cancer-treatment plans via its powerful data-processing ability, sometimes recommended inappropriate treatments; reports attribute this to limitations of training data and input-data quality (22).

The decision process of AI models is often opaque, making it complex to verify the reliability of AI-proposed drug designs or research results. This “black-box” characteristic may cause researchers difficulty in understanding the R&D logic and make regulators struggle to assess compliance and scientific validity (23). When AI models are used to predict the probability of genetic diseases, although such predictions can theoretically aid early intervention, they also raise ethical questions about whether patients should be told these future health risks (24). If predictions are inaccurate, unnecessary anxiety or wrong medical decisions may result. One study indicated that knowing one’s disease susceptibility may have psychological impacts, and, when no practical intervention exists, such knowledge may do more harm than good (25).

### 3.2 Ethical misconduct in human behavior

#### 3.2.1 Ethical breach of the pre-clinical dual-track verification mechanism

As AI technology compresses the drug-development cycle from the traditional 10 years to a few years or even months, the systemic absence of long-term toxicity observation is becoming a core hidden danger threatening drug safety. In traditional R&D systems, toxicity monitoring lasting several years is key to capturing delayed adverse reactions, genetic toxicity, and cumulative metabolic risks. The AI-driven R&D model, pursuing efficiency, often replaces part of long-term experiments with virtual models or shortens observation periods in pre-clinical trials, which may obscure adverse effects in long-term metabolism or intergenerational transmission.

Just as the thalidomide tragedy revealed the limitations of extrapolating animal experiments to humans, it also sounds a warning for AI-accelerated R&D—although AI improves efficiency by simulating animal physiology and compressing intergenerational experiments, advancing subsequent stages merely because no short-term abnormalities appear in first-generation trials may neglect genetic toxicity and delayed risks. One difficulty in governing medical AI ethically is that the speed of technological iteration far outpaces updates to ethical norms, causing long-term risk assessment to lag; this, together with the “black-box” nature of AI models, intensifies uncertainty in toxicity prediction (26). In addition, companies such as Google claim to compress the R&D cycle to 2 years or even 6 months; their models rely on historical data and cannot predict unknown reactions of biological systems. The value of the traditional “ten-year cycle” lies in verifying risks through long-term monitoring and intergenerational observation; shortening the cycle with AI may sacrifice key observation windows, laying a hidden danger of chronic toxicity and potentially missing delayed teratogenic risks like the thalidomide incident, violating the “dual-track verification” principle in the pre-clinical evaluation dimension.

#### 3.2.2 Ethical breach of the informed-consent principle in data mining

In recent years, some cases have reflected potential privacy risks of AI and big-data technology, deserving in-depth analysis and discussion. In healthcare, the data-sharing controversy between DeepMind and the United Kingdom’s National Health Service (NHS) is typical (27); ostensibly aimed at predicting acute kidney injury with AI, it actually manifested three ethical defects in data sharing:

During the development of the “Streams” app by DeepMind and NHS, three major ethical irregularities in processing 1.6 million patients’ medical data directly violated the principle of “substantial informed consent in the data-mining stage”:

First, the NHS data-use consent form did not explicitly state that data would be used to train AI algorithms for a commercial entity, using the vague wording “may be used for research” to evade the duty of explanation, essentially violating Article 22 of the Declaration of Helsinki, which requires that research purposes be clearly explained.

Second, de-identification retained correlated fields such as timestamps of clinical events and disease codes; empirical research at the University of Cambridge showed that combining the temporal features of medical records with public data could re-identify 15% of patients, clearly violating Article 4 of the 1998 Data Protection Act (UK), which legally requires anonymization to achieve non-identifiability.

Third, the project deliberately bypassed review by an independent Research Ethics Committee (REC) and did not follow the NHS Research Ethics Framework’s requirement for a full ethical review of commercial data research, rendering the review process void.

After exposure, the incident aroused systemic public concern: an investigation by The Guardian confirmed that NHS’s legal team was informed unilaterally only 3 months before data transfer and that patients were never notified; scholars at University College London wrote in Nature that the case sets a dangerous precedent for the commercial misuse of public medical data, etc. In 2018, the Information Commissioner’s Office (ICO) ruled that the case violated the Data Protection Act and forced the establishment of a transparent data-sharing platform. NHS’s 2019 “Guidelines on Commercial Use of Health Data” established a regulatory mechanism of “explicit consent + third-party data trust.” As the first typical medical-data violation case after GDPR implementation, it has been officially included in the annotation database for Article 9 (processing of sensitive data) of the GDPR, becoming a landmark precedent in global medical-AI data governance.

In drug research and development, researchers likewise need to handle large amounts of patient data—including genetic information, personal and family medical histories, and lifestyle habits. Such data help researchers better understand disease mechanisms and patient response differences to design more targeted drugs. However, they also raise significant ethical issues: how to tap the data’s potential while protecting patient privacy from infringement. Similarly, 23andMe’s cooperation with pharmaceutical companies raised privacy concerns (28); accumulating massive genetic data via consumer genome services, it signed a 2018 agreement with GlaxoSmithKline to share its genetic database for new drug development. Although the statement included privacy protection, the public worried that personal genetic information might be used for commercial profit. 23andMe did not obtain separate ethical consent for “population-gene frequency analysis,” thus violating the informed-consent standard in the data-mining dimension. This risk is more pronounced in cross-border data transfer: for instance, a multinational drug company’s Hong Kong subsidiary circumvented approval procedures, exposing the gap between group genetic-data regulation and framework requirements.

Human actors also exploit regulatory loopholes: in low-cost trial regions such as Cambodia and South Africa, pharmaceutical companies often make use of weak local ethical-review capacity and scarce medical resources to conduct clinical trials that could not pass ethical review in developed countries, essentially shifting R&D risk artificially. A typical case showed that, in a Phase III trial of an anti-HIV drug in African children, the control group still used outdated AZT monotherapy, artificially enlarging the mortality difference between the test and control groups; this geographical bias essentially shifts R&D risk to developing countries. An even more concealed double standard exists in pediatric drug use: parents in Europe and the U. S. generally oppose including their own children in new-drug trials but tacitly permit pharmaceutical companies to recruit poor-family children in India or Brazil as subjects, forming an “ethical exemption” mind-set of “testing drugs on other people’s children,” which clearly violates the Declaration of Helsinki’s principle of “risk–benefit balance.”

#### 3.2.3 Delay in domestic regulation and systems

The application of artificial intelligence (AI) and big-data technology in drug R&D is a double-edged sword: while increasing efficiency and bringing unprecedented opportunities, it also brings complex ethical responsibilities and regulatory demands. In traditional drug-development processes, every step of decision-making can be traced back to specific researchers or teams. However, when AI is involved in decision-making—especially when AI automatically performs data analysis and pattern recognition—determining the attribution of responsibility becomes particularly difficult (29). For example, in the aforementioned case of IBM Watson: when it recommended inappropriate treatment methods, if adverse events occurred, should responsibility lie with the AI developers, users, or the AI itself? This not only highlights the challenge of responsibility attribution in AI-assisted medical decisions and drug design but also reflects the complexity of regulating such technological applications.

The use of AI and big data must conform to existing pharmaceutical regulations and ethical norms. However, many existing regulations are not fully adapted to the characteristics of AI technology (30). For example: when AI models process patients’ privacy data, a new problem arises as to how to ensure data confidentiality or patients’ privacy rights. In addition, the application of AI in drug R&D may involve new risks, such as the algorithmic bias mentioned earlier, which may lead to unequal distribution of drug efficacy (1). How to make it fairer and better serve all patients is also a current regulatory challenge.

In the data-mining dimension, regarding the regulatory gap in group genetic data, Article 36 of GCP clearly requires that “clinical-trial data shall be authentic, complete, and traceable,” yet regulatory arbitrage in cross-regional trials is common. A typical case showed that an anticancer drug completed Phase I clinical trials at a tertiary hospital in Shanghai but, without completing the required three-month safety follow-up, transferred the remaining cases to a hospital in Xi’an to continue Phase II trials via “data slicing,” using geographical dispersion to obscure signals of hepatotoxicity. Although China has issued the “New-Generation Artificial-Intelligence Development Plan,” proposing a three-step strategy to establish an AI-ethics legal system, specific standards are still lacking for cross-border transmission of group genetic data, algorithmic bias, and other issues in drug R&D (31). This operation exploits the decentralization of ethical reviews in domestic multi-center trials—each hospital’s ethics committee only reviews local data and lacks a cross-regional data-linkage verification mechanism, resulting in “patching data from different locations” as a gray method for evading long-term toxicity monitoring. According to the National Medical Products Administration’s 2023 spot-inspection report, 28% of clinical-trial institutions had such problems, and the violation rate in cross-regional trials was 3.2 × that of single-center trials.

Compared with Article 9 of the EU GDPR, which strictly regulates the handling of genetic data, China’s current regulations have obvious institutional gaps. The “Regulations on Administration of Human Genetic Resources” cover only “human genetic-resource materials” and lack regulation of anonymized genetic-sequencing data in cross-border transmission; a multinational pharmaceutical company once bypassed approval through a Hong-Kong subsidiary and was publicly reported by the Ministry of Science and Technology. The “Personal Information Protection Law” classifies “biometric data” as sensitive information but does not explicitly define the legal status of “group genetic data”; a bioinformatics company was interviewed by the Cyberspace Administration for using genetic data of Northwestern populations to train models without group-level ethical consent. The “Data Security Law” does not specify whether “group genetic data” constitute critical data; in practice, drug companies often transmit data abroad in the name of “research data,” such as a Chinese-American joint-venture drug company submitting unapproved Han-Chinese gene-frequency data to the U. S. FDA.

In the pre-clinical and patient-recruitment dimensions, current traditional regulatory means may find it difficult to handle group-data risks in the AI era. First, this is reflected in weak cross-regional data-traceability capability: domestic clinical-trial data platforms have not yet achieved national networking, and the NMPA inspection system cannot compare patient-enrollment data across institutions in real time, making it hard to identify “the same patient enrolled in different cities.” Second, it is reflected in the absence of an algorithm-audit mechanism: when drug companies use AI models to analyze group genetic data, regulators lack technical means to verify whether algorithms are biased. In 2024, an innovative drug company’s AI-assisted diagnostic model was found to have a diagnostic accuracy for Tibetan people 35% lower than for Han people; the NMPA required supplementary ethnic-group data, but current regulations have no technical standards for algorithmic fairness.

The deep reason for this regulatory loophole is that China’s drug-development regulation still follows an “individual-safety-oriented” institutional logic, while the application of AI and big-data technology has expanded the risk dimension to group genetic safety and bioethics. As the NMPA’s 2024 “White Paper on AI Drug-Development Supervision” points out: “It is necessary to build a new three-in-one regulatory framework of ‘data-algorithm-ethics,’ include group-genetic-data protection in the key points for revising the ‘Regulations on Administration of Human Genetic Resources,’ and establish a blockchain certificate-deposit system for cross-regional clinical-trial data, tackling regulatory difficulties through both technical traceability and institutional improvement.”

#### 3.2.4 Cross-national implementation dilemmas under the three-dimensional ethical-evaluation principle

In the context of global cooperation in drug development, when AI technology accelerates cross-border drug R&D, the biological differences of animal-model extrapolation and the institutional vacuum in international responsibility allocation are forming a superimposed double risk.

Biological uncertainty exists in extrapolating animal models: for example, anti-fibrotic drugs targeting TGF-β can reverse liver fibrosis in rat models, but clinical Phase III had to be terminated for inducing human cholangiocarcinoma, because the TGF-β-signal threshold of rat hepatic-stellate cells is three times that of humans. When AI accelerates R&D by simulating animal physiology, such risks may be amplified: a multinational pharmaceutical company advanced an international multi-center trial of a lung-cancer drug based on AI-predicted mouse-toxicity data, but the model did not include the human-specific CYP2F1 enzyme metabolic pathway in lung epithelial cells, resulting in interstitial pneumonia in American subjects.

International cooperation currently still has a responsibility gap, especially manifested in regulatory arbitrage and fragmented standards. When drug development crosses national borders, cross-national use of animal-experiment data is often accompanied by vague responsibility. For example, in a Sino-U. S. collaborative gene-therapy drug project, safety evaluation in primate experiments was completed by the U. S. team; the Chinese team advanced Phase I trials based on AI-simulation data. However, because the two sides had different standards for monkey-kidney toxicity thresholds, when irreversible kidney damage occurred in Chinese subjects, Chinese and American regulators delayed handling due to disputes over responsibility allocation.

This institutional gap may stem from international differences in animal-experiment standards and liability exemptions for AI-model intellectual property. The FDA requires non-rodent animal experiments to last at least 6 months, while the NMPA may accept three-month data for certain orphan drugs. A Sino-European orphan-drug collaboration used this difference, obtaining EU approval with six-month canine data while submitting only three-month monkey data in China; it was eventually recalled because Chinese patients experienced cardiotoxicity already observed in dog models. Moreover, a Korean pharmaceutical company exported an AI-assisted diagnostic system to an African nation; its animal-experiment data were based on Korean macaques, but the model was not adjusted for genetic polymorphisms of African green monkeys, increasing the misdiagnosis rate and exposing a responsibility vacuum in cross-border technology export.

The essence of this responsibility ambiguity is the institutional imbalance of “localizing risks while internationalizing profits” in the global R&D system. As the 2023 CIOMS “Guidelines on Ethical AI Drug Development” state: when animal-experiment data are transmitted cross-border by AI and used for human decisions, a full-chain responsibility-traceability mechanism of “data source—algorithm logic—clinical application” must be established to avoid shifting biological-difference risks to regions with weak regulation. Solving this problem requires not only unifying cross-species extrapolation standards for animal models but also constructing an international shared-responsibility mechanism for AI R&D under the WHO framework.

## 4 Countermeasure suggestions

Responsible innovation is an innovation strategy that takes ethics, social responsibility, and sustainability as its core, aiming to ensure that technological progress enhances human well-being while minimizing negative impacts on the environment and society. Applying the principles of responsible innovation can promote the development of more transparent and fair AI systems (32), strengthen ethical standards of data management, and drive the establishment of more comprehensive ethical-review mechanisms. In addition, by promoting interdisciplinary cooperation, bringing together ethicists, legal experts, and technologists to study and solve complex problems brought by new technology, this approach not only enhances public trust in AI-assisted drug development and treatment plans but also helps ensure that technological innovation proceeds within moral and legal frameworks, enabling its achievements to bring maximum benefit to society.

### 4.1 Strengthening ethical-review and supervision frameworks

#### 4.1.1 Implementation of informed consent in the data-mining stage

In the data-mining dimension, it is necessary to examine whether group-genetic-data collection meets the standards of “explicit consent and third-party data trust,” while simultaneously enhancing algorithmic transparency and explainability—this is a key link in strengthening ethical-review and supervision frameworks. The EU “Ethics Guidelines for Trustworthy AI” propose that AI systems should meet seven requirements, including “human agency and oversight” and “privacy and data governance,” providing a reference for improving algorithmic transparency in drug R&D (33). Decisions made by AI algorithms in drug design, screening, and clinical-drug selection can be extremely complex, but the bases for these decisions must be explicable and reviewable to avoid a “black box” (34). This requires researchers not only to disclose algorithm design and operation mechanisms but also to ensure that algorithm-decision logic is traceable, allowing third-party auditing of AI decision processes. Standardized algorithm-audit procedures can be developed to ensure that the algorithm’s application undergoes continuous effectiveness evaluation and risk monitoring. In addition, an internationally accepted ethical-review mechanism for sharing group genetic data should be established, requiring multinational drug companies to obtain approval from multi-party ethics committees when collecting, transferring, and using human genetic data, and clarifying data sovereignty and cross-border flow rules; at the same time, drawing on GDPR experience, the informed-consent standards for group genetic data should be refined to eliminate “genetic colonialism” risks.

#### 4.1.2 Strengthening dual-track verification in pre-clinical research

The AI technology compresses the drug-development cycle from the traditional 10 years to 2 years. While simulating animal physiology and accelerating intergenerational experiments to achieve efficiency improvement, it exposes core contradictions of insufficient long-term toxicity observation and reduced accuracy in extrapolating animal models to humans, such as the tragedy in which animal experiments failed to predict human teratogenicity in the thalidomide incident—risks that may be amplified by AI acceleration.

Therefore, a mandatory dual-track verification mechanism and long-cycle observation system must be implemented: on one hand, AI-designed compounds must undergo “virtual-model prediction + actual animal experiments”—the virtual model uses AI algorithms to simulate drug mechanisms and metabolism, providing preliminary risk assessment, while actual animal experiments verify predictions in real organisms, compensating for the virtual model’s limitation in simulating biological complexity; on the other hand, in high-risk key areas such as cardiovascular and neuropsychiatric drugs, clinical studies must set at least a one-year human follow-up and establish dynamic monitoring databases, tracking the safety and effectiveness of drugs post-marketing to avoid missing chronic toxicity due to short observation periods. Furthermore, a rigorous review process must be established, requiring developers to submit detailed dual-track verification reports, with regulators conducting regular spot checks to ensure the authenticity and reliability of pre-clinical data, thereby safeguarding safety before drugs enter clinical trials.

#### 4.1.3 Improving transparency in the patient-recruitment stage

To improve ethical compliance in patient recruitment, double auditing of AI enrollment algorithms can be implemented. First, sample proportions of each major ethnic group in the algorithm must be ≥ the demographic proportion of the target population; IBM AIF360 and other professional tools can be used to detect algorithmic bias, ensuring risk-prediction deviation among ethnic groups < 5%, and regular public audit reports plus detailed algorithm-process descriptions guarantee patients’ right to know. Second, a “biological-difference compensation factor” can be introduced into AI drug-development models: by integrating multi-species physiological parameters and metabolizing-enzyme activity data, and constructing a biomimetic virtual experimental environment with organ-on-a-chip and organoid technology, after calibration, predictive deviation between animal-experiment results and human response can be <5%, thereby improving AI-prediction reliability at the data source and promoting fairness and scientificity in clinical-trial enrollment.

### 4.2 Protecting patients’ data privacy

The involvement of AI and big data in healthcare covers large amounts of sensitive patient data; protecting data privacy thus becomes an urgent issue. Within a responsible-innovation framework, ensuring patient data privacy is not only a legal and ethical requirement but also embodies technological progress and social responsibility. Privacy protection of medical AI must adopt a “technology + system” dual path: for example, differential-privacy technology can retain statistical value while hiding individual information in data sharing, and blockchain technology can realize data traceability and access control (35).

Protecting patient data privacy must start from the data-collection source. At every stage of data collection, storage, and processing, relevant legal norms must be strictly observed, such as the EU GDPR and the U. S. HIPAA. These laws provide a strong protection framework for personal data, ensuring the reflection of personal privacy rights during data processing. For example, researchers must ensure that patients fully understand that their data will be used for research purposes and give explicit consent.

To further strengthen privacy protection, high-standard technical means are needed: encryption during transmission to prevent unauthorized access and strict access control to ensure only relevant personnel can access sensitive data. In addition, anonymization can allow data use in research without revealing personal identity, reducing the risk of information leakage (36). Beyond technical and legal support, a culture valuing data-privacy rights must be cultivated across the drug-development field; every participant, from researchers to management, must receive data-protection training, understand its importance (37), and implement this principle in daily work.

### 4.3 Improving algorithmic fairness and reliability

Improving algorithmic fairness and reliability is not only ethically justified but also an important guarantee for enhancing research accuracy and efficiency. Building multi-source heterogeneous datasets and establishing long-term audit mechanisms are core paths for improving algorithmic fairness. On one hand, balanced data covering different regions, races, ages, and disease types must be collected, especially increasing the proportion of rare-disease and special-group data to avoid algorithmic bias at the source. On the other hand, independent third-party institutions should establish a normalized fairness-evaluation mechanism; audit content includes data bias, decision logic, and group-impact differences, linking audit results to R&D qualification dynamically—enterprises failing audits would face rectification deadlines or project-qualification suspension. This also requires a cross-disciplinary collaborative mechanism involving statisticians, clinicians, drug R&D personnel, and ethicists (38) to identify algorithm-bias risks from multiple perspectives. Problems found must be promptly adjusted and optimized to avoid long-term accumulated bias affecting fairness and scientific validity of R&D results.

## 5 Conclusion

The development and application of large AI and big-data models bring more innovative possibilities and opportunities to drug R&D but also trigger many ethical challenges. As large AI and big-data models become increasingly integrated into the performance and development of new drugs, management of pharmaceutical ethics becomes ever more important, and research on responsible innovation in drug R&D will become ever stricter. At present, China’s legal and regulatory system for protecting data-privacy rights, safeguarding subjects’ rights, and realizing ethical values in drug R&D is still imperfect; government, society, and individuals all need to strengthen ethical-protection awareness and improve moral cultivation. By embedding the three-dimensional ethical-evaluation framework of “data mining — pre-clinical — patient recruitment,” this paper constructs a comprehensive system of pharmaceutical-ethics governance. The application of large AI and big-data models to responsible innovation in drug R&D should always take the protection of human life and health as its purpose, pursuing the coordinated development of technological progress and ethical cultivation.
